# Identification of Esters as Novel Aggregation Pheromone Components Produced by the Male Powder-Post Beetle, *Lyctus africanus* Lesne (Coleoptera: Lyctinae)

**DOI:** 10.1371/journal.pone.0141799

**Published:** 2015-11-06

**Authors:** Titik Kartika, Nobuhiro Shimizu, Tsuyoshi Yoshimura

**Affiliations:** 1 Research Centre for Biomaterials, Indonesian Institute of Sciences, West Java, Indonesia; 2 Research Institute for Sustainable Humanosphere, Kyoto University, Gokasho, Uji, Japan; 3 Faculty of Bioenvironmental Science, Kyoto Gakuen University, Kameoka, Japan; INRA-UPMC, FRANCE

## Abstract

*Lyctus africanus* is a cosmopolitan powder-post beetle that is considered one of the major pests threatening timber and timber products. Because infestations of this beetle are inconspicuous, damage is difficult to detect and identification is often delayed. We identified the chemical compounds involved in the aggregation behavior of *L*. *africanus* using preparations of crude hexanic extracts from male and female beetles (ME and FE, respectively). Both male and female beetles showed significant preferences for ME, which was found to contain three esters. FE was ignored by both the sexes. Further bioassay confirmed the role of esters in the aggregation behavior of *L*. *africanus*. Three esters were identified as 2-propyl dodecanoate, 3-pentyl dodecanoate, and 3-pentyl tetradecanoate. Further behavioral bioassays revealed 3-pentyl dodecanoate to play the main role in the aggregation behavior of female *L*. *africanus* beetles. However, significantly more beetles aggregated on a paper disk treated with a blend of the three esters than on a paper disk treated with a single ester. This is the first report on pheromone identification in *L*. *africanus*; in addition, the study for the first time presents 3-pentyl dodecanoate as an insect pheromone.

## Introduction

Powder-post beetles are common pests of dry wood and can digest the wood components of sapwood, primarily starch, producing a fine powdery frass [1]. *Lyctus* is a powder-post beetle belonging to the subfamily Lyctinae in the family Bostrichidae [1]. Many species in this beetle grouping are cosmopolitan and are commonly found in Africa [2], Southeast Asia [3], North America [1], Europe [4], Australia [5] and Japan [6] as either native or introduced species.

In Japan, *Lyctus africanus* is increasing in number and is currently widespread in many prefectures [7]. The insect is considered to be a major pest that threatens timber and timber products, including plywood [4], furniture, doors, and ceilings [8].

Some monitoring techniques have been developed to regularly inspect insect populations. Insect monitoring by trapping has been widely used in pest management [9]. Early detection of infestation may help minimize damage and improve prevention. Recording insect numbers over time allows precise monitoring on insect populations. Insects can be attracted to food baits, light sources, or color traps. One preliminary study of *L*. *brunneus* found that both male and female beetles were intensively attracted by female odor [10]; however, there was no further information confirming that this attraction was due to a pheromone. Given that species in the *Lyctus* group are third on the list of important wood-destroying pests, following only subterranean and drywood termites, identification of aggregation pheromones and strategies for monitoring and control are needed urgently.

In this study, we describe for the first time the aggregation pheromones and behaviors of adult *L*. *africanus* beetles. Studying the aggregation behavior of *Lyctus* beetles is crucial for understanding the underlying ecological aspects. The behaviors reported here were elucidated using a chemical approach involving the comprehensive screening of candidate compounds produced by *L*. *africanus*. This study will contribute to our understanding of *Lyctus* ecology and aid in the development of monitoring techniques.

## Materials and Methods

### Insect colony

For behavioral and chemical analytical investigations conducted in this study, only adult *L*. *africanus* beetles were used. The beetles were obtained from laboratory sources maintained at the Research Institute for Sustainable Humanosphere, Kyoto, Gokasho, Uji, Japan. This beetle has been maintained in the laboratory for at least 20 years (T. Yoshimura, personal communication). The beetles were reared on an artificial diet consisting of dried yeast (24%, Asahi Food and Health Care, Japan), starch (50%, Nacalai Tesque, Japan), and lauan (*Shorea* spp.) wood sawdust (26%). Glass jars (450 mL) containing beetle cultures were maintained in a sealed chamber at a constant temperature (26°C ± 2°C) and relative humidity (65% ± 10%). Newly emerged adult female and male beetles were sexed and separated under a dissection microscope using apical hairs present along the hind margin of the abdominal segment [2].

### Collection of chemical compound

To collect chemical compounds, whole-body extractions using hexane washes of newly emerged beetles (1–7 days old) were performed by methods similar to those reported for other beetle and millipede groups [11–13]. The newly emerged *Lyctus* are sexually mature upon emergence [2, 14]. Fifty adults of each sex were placed in individual polyethylene test tubes (2-mL self-locking Eppendorf tubes, Germany), 1000μL of hexane was added, and the tubes left were incubated in a constant-environment chamber for 24 h. The extract was transferred into a new empty polyethylene tube for further processing.

### Behavioral bioassay

We conducted bioassays to measure the responses of adult beetles to hexane washes of male and female bodies. The assay used was an adaptation of the dual-choice bioassays [11, 15]. Bioassays were conducted in a closed Petri dish (90 mm in diameter) with a filter paper (90 mm in diameter, Whatman No. 1, GE Healthcare, UK) at the bottom. Two small paper disks (10 mm in diameter, 60 mg, Advantec type 27, Toyo Roshi Kaisha, Japan) were used to receive aliquots of solutions containing either the treatment substance or the control. Each small paper disk in a Petri dish was saturated with 50 μL (equivalent to 2.5 beetles) of the male crude extract (ME) or female crude extract (FE). The same volume of hexane was applied to the control paper disk, and the disks were then air-dried for approximately 1 min before the testing. Each pair of paper disks was placed in a Petri dish, with the disks placed opposite each other 60 mm apart. Prior to observation, the positions of the paper disks in the dishes were randomized. A group of 20 *L*. *africanus* beetles, both males and females, were placed in the middle of the dish as well as at the far edge where the vertical and horizontal surfaces met (S1 Fig). This arrangement in the dishes allowed all beetles to have the same starting point at equal distances from the two filter paper disks. Images of the Petri dishes were recorded at 30-s intervals over a period of 5 min using a video recorder (Sony, model HDR-XR 500 V, Japan). The video tapes were played back to count the number of beetles on each filter paper disk. Beetles settling away from the control and test disks were scored as nonresponders. All observations of beetles were conducted in a dark chamber under red-light illumination with constant temperature and humidity.

### Chemical analysis

GC–MS was performed to identify specific chemical compounds in the crude extracts that induced the aggregation of beetles on the paper disks. The extracts were prepared by immersion of a single adult beetle in hexane (10 μL) for 5 min and were injected into a GC–MS instrument. The instrument used was a Network GC system (6890N; Agilent Technologies, USA) coupled with a mass selective detector (5975 Inert XL; Agilent Technologies) operated at 70 eV. The column used was an HP-5ms capillary column (Agilent Technologies, 0.25-mm I.D. × 30 m, 0.25-μm film thickness). The carrier gas was helium, with a constant flow rate of 1 ml/min. Samples were analyzed in the splitless mode with the temperature programmed to change from 60°C (initially for 2 min) to 290°C at a rate of 10°C/min. The final temperature (290°C) was then maintained for 5 min. The GC–MS data were recorded using Chemstation (Agilent Technologies) with reference to an MS data base (Agilent NIST05 mass spectral library, Agilent Technologies). Column chromatography was performed on a Wakosil silica gel C-200 column with the specified solvents. ^1^H- and ^13^C-NMR spectra were recorded on a Bruker Biospin AC400M spectrometer (400 MHz for ^1^H and 100 MHz for ^13^C) using tetramethylsilane as the internal standard.

### Purification and isolation of male produced compounds

Purification of the chemical compounds was performed by serial extraction. First, ME was prepared in the hexane solvent. One hundred male beetles were transferred into a clear polyethylene tube (a 2-mL self-locking Eppendorf tube) and immersed in 2 ml hexane for 24 h. The resulting extracts were filtered through Whatman No. 1 paper, concentrated in vacuo, and applied to an SiO_2_ column (0.5 g of Wakosil silica gel). The column was successively eluted with hexane, three mixtures of ethyl acetate (EtOAc) in hexane (10%, 20%, and 50%), and EtOAc (5 mL each). All fractions were subjected to GC–MS analyses for identification and were concentrated to 2-ml aliquots for a bioassay to confirm the activity of each fraction.

### Syntheses of three esters

Based on the GC–MS analysis, three esters were identified in ME. The esters were synthesized to confirm their activities on adult *L*. *africanus* beetles. Dodecanoyl chloride (1.0 g, 4.57 mmol) in diethyl ether (5 ml) was added dropwise to a solution of 3-pentanol (0.41 g, 4.66 mmol) and pyridine (0.37 g, 4.66 mmol) dissolved in diethyl ether (10 ml) at 0°C. The mixture was stirred at room temperature for 60 min. After filtration, the eluate was concentrated and then introduced in an SiO_2_ column. Elution with 10% EtOAc in hexane afforded 1.17 g (4.34 mmol, 95%) of 3-pentyl dodecanoate **2** as a colorless oil. GC–MS *t*_R_: 16.95 min. ^1^H NMR (CDCl_3_, δ ppm): 0.88 (t, 9H, *J* = 6.6 Hz, CH_3_), 1.21–1.34 (m, 16H, CH_2_), 1.52–1.64 (m, 6H, CH_2_), 2.31 (t, *J* = 7.2 Hz, 2H,CH_2_CH_2_CO), 4.76 (tt, *J* = 6.8, 5.6 Hz, 1H, OCH(CH)_2_); ^13^C NMR (CDCl_3_,δ ppm): 173.8, 76.4, 34.7, 31.9, 29.6 (× 2), 29.5, 29.3, 29.3, 29.2, 26.5 (× 2), 25,2, 22.7, 14.1, 9.6 (× 2).

The remaining 2 esters, 2-propyl dodecanoate **1** and 3-pentyl tetradodecanoate **3**, were obtained using the same procedure employed in the synthesis of 2-propyl dodecanoate **1** via reactions between the corresponding acyl chlorides and alcohols. 2-propyl dodecanoate **1**: GC–MS *t*_R_: 14.92 min. ^1^H NMR (CDCl_3_, δ ppm): 0.88 (t, 3H, *J* = 6.6 Hz, CH_2_CH_3_), 1.25 (d, 6H, *J* = 4.0 Hz, CH(CH_3_)_2_), 1.21–1.33 (m, 16H, CH_2_), 1.61 (m, 2H, CH_2_CH_2_CO), 2.25 (t, *J* = 7.6 Hz, 2H,CH_2_CH_2_CO), 5.00 (septet, *J* = 6.4 Hz, 1H, OCH(CH_3_)_2_); ^13^C NMR (CDCl_3_, δ ppm): 173.5, 67.3, 34.8, 31.9, 29.6 (× 2), 29.5, 29.3, 29.3, 29.1, 25.1, 24,7, 22.7, 21.9 (× 2). 3-pentyl tetradecanoate **3**: GC–MS *t*_R_: 18.95 min. ^1^H NMR (CDCl_3_, δ ppm): 0.88 (t, 9H, *J* = 6.6 Hz, CH_3_), 1.21–1.33 (m, 20H, CH_2_),1.52–1.64 (m, 6H, CH_2_), 2.29 (t, *J* = 7.6 Hz, 2H,CH_2_CH_2_CO), 4.76 (tt, *J* = 6.8, 5.6 Hz, 1H, OCH(CH)_2_); ^13^C NMR (CDCl_3_, δ ppm): 173.8, 76.3, 34.7, 31.9, 29.7, 29.6 (× 2), 29.6, 29.5, 29.3, 29.3, 29.2, 26.5 (× 2), 25.2, 22.7, 14.1, 9.6 (× 2).

### Quantitative determination of three esters

To determine the quantity of each ester in a male *Lyctus* beetle, a calibration curve was constructed for each compound. The curve was obtained by correlating the GC–MS response data of ME with each concentration of three standard solutions. ME was prepared by dipping one male *L*. *africanus* beetle into hexane (10 μL), followed by extraction for 5 min. The extract was collected with a 10-μL microsyringe and subjected to GC–MS analysis (*n* = 9 trials). A synthetic sample of each ester (2-propyl dodecanoate **1**, 3-pentyl dodecanoate **2**, and 3-pentyl tetradecanoate **3**) was diluted with hexane. The following concentrations were prepared: 5, 10, and 25 ng/μL; a 200 ng/μL solution was also prepared for the major compound (**2**). A calibration curve was then constructed.

### Aggregation activity of natural and synthetic esters

The behavioral activity of ME was compared with the responses to the synthetic compounds. To evaluate the behavioral activity of the crude extract as a criterion for the isolation and identification of the chemical compounds, the synthetic compounds were also tested using a dual-choice laboratory bioassay to show the aggregation behavior of *L*. *africanus*. First, the activity of a single component was measured, followed by the blended compound activity to determine whether synergism occurred with the mixture.

#### Aggregation activity of single ester

Adult male and female beetles were exposed to the identified esters, single compounds **1**–**3**, to assay the function of each compound in the aggregation behavior of *L*. *africanus*. The bioassays were performed in closed Petri dishes using a dual-choice method similar to that of the preceding bioassays. Synthetic compounds **1**–**3** were prepared in serial doses of 2, 20, 200, 400, and 800 ng/disk and presented to both male and female beetles.

#### Aggregation activity of ester blends

Two blends mixing the three esters were prepared to evaluate the biological activity of blends. Blend A reproduced the natural proportions of the esters **1**, **2**, and **3** (20:300:20) as they were measured in the extracts of male *L*. *africanus*; in blend B, the three esters were mixed in equal proportions 300:300:300. The two blends were tested on behavior as described before.

### Statistical analysis

Ten replicates were performed for each treatment combination (crude extract, fractions, single compounds, and blend compounds). The counts of beetles preferring either treated or untreated (control) paper disks were scored after 5 min of observation and analyzed for differences. To determine the responses of beetles to the extracts, an aggregation index (AI) was calculated as *(T − C)/N*, where *T* is the number of beetles located on the treated paper disk, *C* is the number of beetles located on the control disk, and *N* is the total number of beetles used in the bioassay [16–19]. The index value ranged from +1 to −1. Only a positive value of AI corresponded to an aggregation response. The index values of the responses were subjected to one-way analysis of variance, followed by Tukey’s honest significant difference (HSD) test (α = 0.01), or Student’s *t* test (JMP version 9; SAS Ins.).

## Results

### Bioassay of the crude extracts

Based on the laboratory tests and recorded visual observations, *L*. *africanus* showed aggregation behavior. Within minutes of being released in Petri dishes, the adults exhibited searching behavior, moving around the dish, and finally formed a group or cluster. Treatment replicates for the adults included crude extracts from both male and female beetle in three combinations: ME vs. control, FE vs. control, and ME vs. FE. The beetles aggregated immediately on the preferred disk. Both female and male beetles showed a significantly higher preference for ME-treated paper disks than for control paper disks (S1 Table). The responses of the beetles were expressed in terms of AI, shown in Table 1. AI indicated that both female and male beetles preferred ME-treated paper disks over FE-treated disks (female: *F* = 50.72, *df* = 29, *P* < 0.0001; male: *F* = 7.50, *df* = 29, *P* < 0.0001). However, female beetles showed a higher preference than male beetles for ME-treated paper disks (Student’s *t* test, *P* < 0.05). FE-treated paper disks were not preferred over control by males nor female beetles. This result suggests that the ME contained specific compounds that triggered the aggregation behavior in *L*. *africanus*.

**Table 1:** Response of adult *Lyctus africanus* beetles to paper disks treated with the male crude extract (ME), female crude extract (FE), or control (50 μL = equivalent to 2.5 beetles), presented by the aggregation index value (*N* = 20; *n* = 10).

Tested beetles	Treatment		
	ME vs. Control	FE vs. Control	ME vs. FE
♀	0.62 ± 0.04^a(^ * ^)^	0.07 ± 0.06^b(ns)^	0.50 ± 0.03^a(^ * ^)^
♂	0.36 ± 0.04^a(^ * ^)^	0.04 ± 0.02^b(ns)^	0.28 ± 0.06^ab(^ * ^)^

Notes: Different letters indicate significant difference (*p* < 0.01, Tukey’s HSD test) in the aggregation index (mean ± SE) of female and male beetles within treatments. Letters in parentheses refer to the comparison between female and male beetles in the same treatment (Student’s *t* test).

*: Significant; ns: not significant.

### Chemical analysis

**Fig 1:**
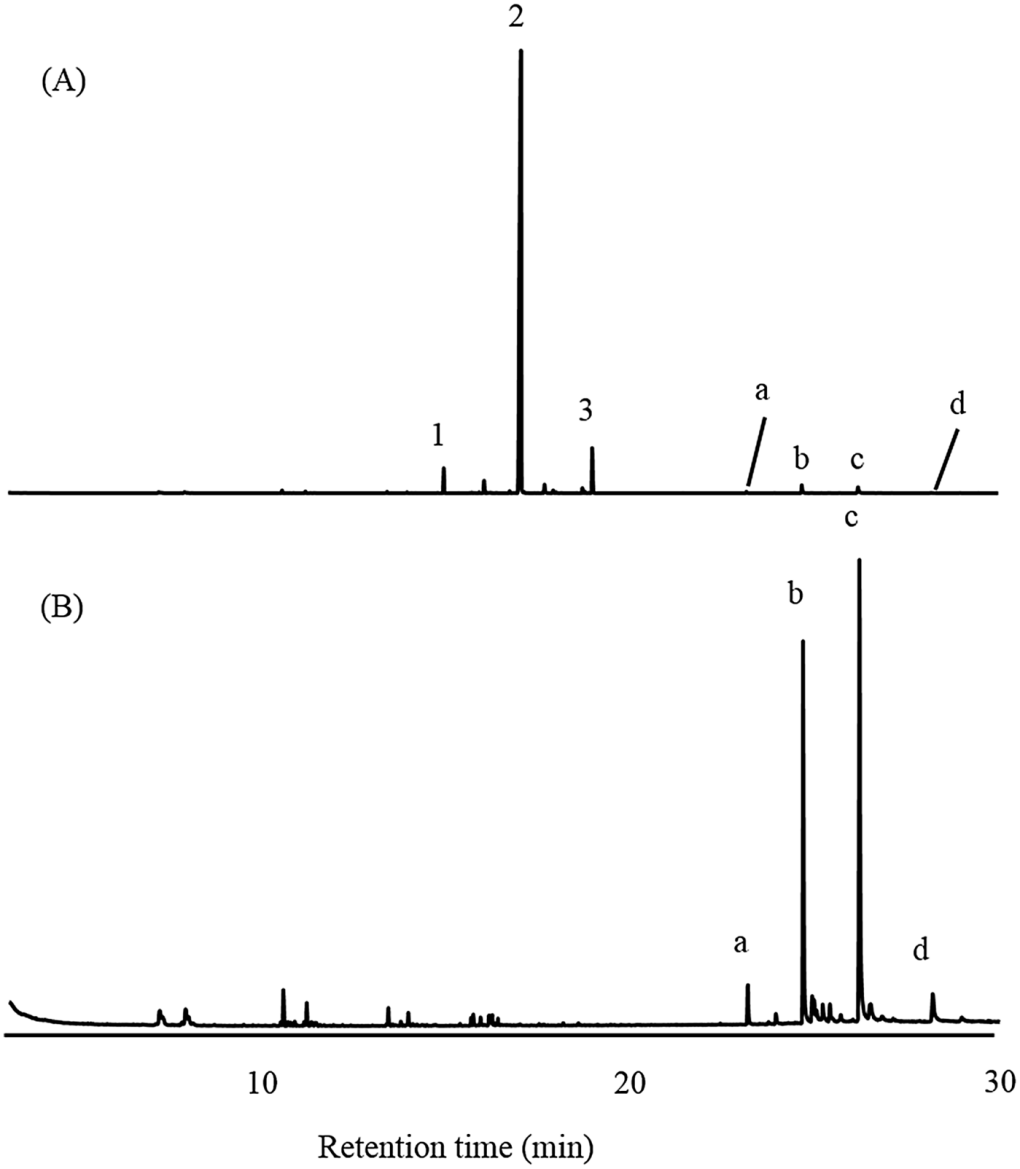
Gas chromatographic comparison of crude extracts from (A) an adult male and (B) an adult female; four peaks (a, b, c and d, identified as C_25_, C_27_, C_29_, and C_31_, respectively) were found in both extracts, whereas three peaks (compounds 1, 2 and 3) were found to be male-specific components.

ME and FE were compared using GC–MS (Fig 1). Three peaks (**1**, *t*_R_ 14.92 min; **2**, *t*_R_ 16.95 min; and **3**, *t*_R_ 18.95 min) were detected in ME. In contrast, no trace of the corresponding peaks was found in FE. Other compounds (pentacosane, C_25_; heptacosane, C_27;_ nonacosane, C_29_, and hentriacontane, C_31_) were present in small amounts in male beetles but in larger amounts in female beetles.

### Purification and bioassay for the aggregation pheromone

Crude extracts from 100 adults were separated on an SiO_2_ column into five fractions. Each fraction was subjected to GC–MS. The male-specific components (**1**–**3)** were detected only in the fraction eluted with 10% EtoAc in hexane. To assess pheromonal activity, all fractions were bioassayed using a method similar to that of the previous bioassay involving crude extracts, as summarized in Table 2. Compared with the other fractions, the 10% EtoAc in hexane fraction elicited a stronger aggregation behavior in adult *L*. *africanus* beetles, as indicated by the AI values of the adult beetles, particularly females (female: *F* = 96.08, *df* = 49, *P* < 0.0001; male: *F* = 11.77, *df* = 49, *P* < 0.0001). There was a significant difference in the number of both female and male beetles that aggregated on the paper disk treated with the 10% EtoAc in hexane fraction (S2 Table).

**Table 2:** Response of adult *Lyctus africanus* beetles to paper disks treated with fractions of the male crude extract (50 μL = equivalent to 2.5 beetles), presented by the aggregation index value (*N* = 20; *n* = 10).

Tested beetles	Fractions				
	Hexane	10% EtoAc in hexane	20% EtoAc in hexane	50% EtoAc in hexane	EtoAc
♀	−0.04 ± 0.02^b(ns)^	0.50 ± 0.03^a(^ * ^)^	0.07 ± 0.02^b(ns)^	0.02 ± 0.01^b(^ * ^)^	0.03 ± 0.02^b(ns)^
♂	−0.07 ± 0.02^c(ns)^	0.18 ± 0.04^a(^ * ^)^	0.10 ± 0.04^ab(ns)^	−0.04 ± 0.02^bc(^ * ^)^	−0.02 ± 0.03^bc(ns)^

Notes: Different letters indicate significant differences in the aggregation index of male and female beetles within treatments (Tukey’s HSD test). Letters in parentheses refer to the comparison between female and male beetles for the same fraction (Student’s *t* test).

*: Significant; ns: not significant.

### Identification of the three esters

**Fig 2:**
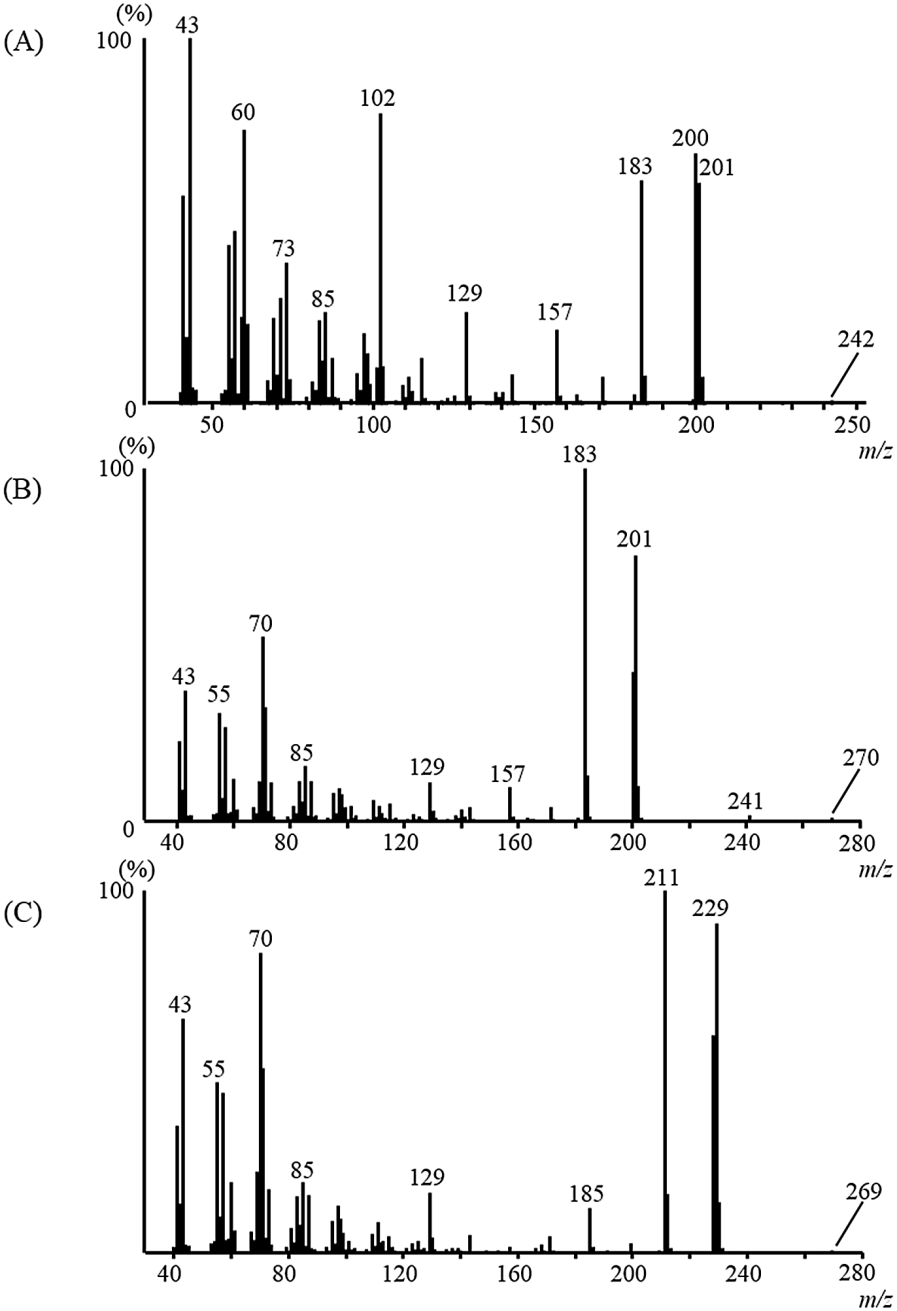
Mass spectra of natural ester compounds present in the male crude extract: (A) 1; (B) 2; and (C) 3.

**Fig 3:**
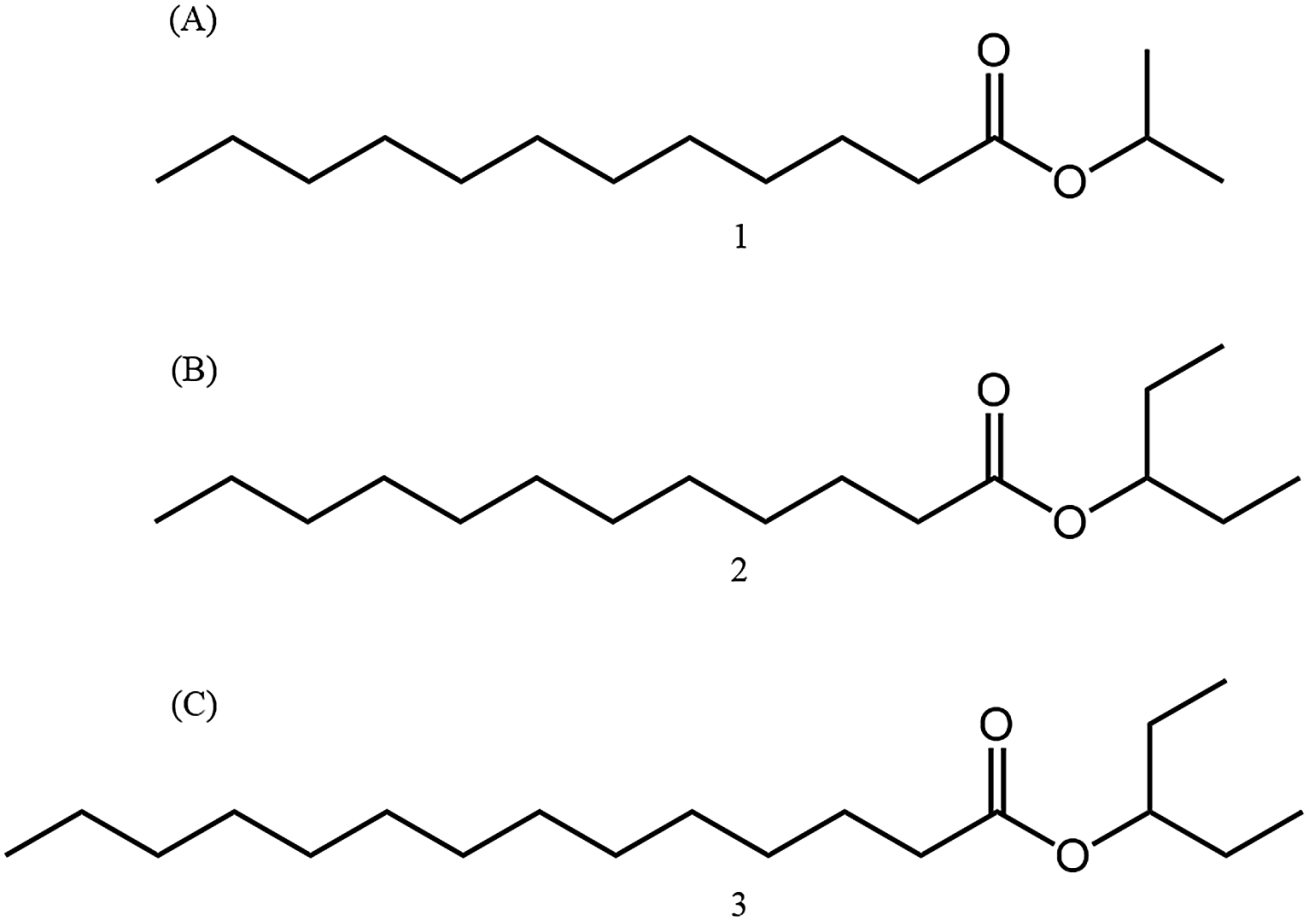
Chemical structures of compounds (A) 1; (B) 2; and (C) 3.

Compound **2** was the most abundant product in the 10% EtoAc in hexane fraction, as indicated by the M^+^ ion at *m/z* 270 and the base ion at *m/z* 183, with a diagnostic ion at *m/z* 201 (Fig 2B). The latter ion was the second most intense ion, indicative of a fragment derived from a C_12_-fatty acid moiety. The base ion recorded at *m/z* 183 was the dehydrated *m/z* 201 ion. The third most intense ion at *m/z* 70 (57%) was found to be derived from a C_5_-alcohol moiety. The mass spectrum of compound **2** resembled that of *n*-pentyl dodecanoate (*n*-pentyl laurate), although the GC *t*_R_ of compound **2** (16.95 min) was shorter than that of *n*-pentyl dodecanoate (17.52 min) [13]. These findings revealed an isomeric relationship between the two compounds and suggested that compound **2** was a dodecanoate of a branched alcohol. In addition, a fatty ester containing an *n*-pentyl alcohol moiety showed a fragment ion of *m/z* 70 as the base ion [compound **4** (*n*-pentyl dodecanoate) and **6** (*n*-pentyl isotridecanoate)] [13]. Four monobranched candidates were prepared as candidate structures for the comparison of the GC *t*_R_ to that of compound **2**. 2-Pentyl dodecanoate (2-PD), 2-methylbutyl dodecanoate (2-MBD), and 3-methylbutyl dodecanoate (3-MBD) were synthesized using the same procedure employed in the synthesis of 3-pentyl dodecanoate **2** via a reaction between the corresponding alcohols and dodecanoyl chloride. As shown in S2A Fig, the four isomers showed different chromatographic behaviors on the GC capillary column (HP-5 ms). Synthetic 3-pentyl dodecanoate yielded a GC *t*_R_ and mass spectrum that were identical to those of natural product **2** (S2B Fig). Compound **2** was accordingly identified as 3-pentyl dodecanoate (Fig 3B).

The diagnostic ion of compound **1** recorded at *m/z* 201 was identified as that of a C_12_ fatty acid, as mentioned above (Fig 2A). The ester containing a 2-propyl alcohol moiety showed fragment ions of *m/z* 43 and *m/z* 60 as the base and diagnostic ions, respectively. Accordingly, compound **1**, which showed an M^+^ ion at *m/z* 242, was identified as 2-propyl dodecanoate **1** (14.92 min). Authentic compound **1** gave the same GC *t*_R_ and mass spectrum as natural product **1**. The fragment ion at *m/z* 229 of compound **3** was comparable to that of a C_14_-fatty acid moiety (Fig 2C). The base ion recorded at *m/z* 211 was the dehydrated *m/z* 229 ion. The diagnostic ion at *m/z* 70 was similar to that of compound **2**, which suggested that **3** was an ester containing a 3-pentyl alcohol moiety. The structure of **3** was thus assigned as that of 3-pentyl tetradecanoate. Authentic compound **3** gave the same GC *t*_R_ and mass spectrum as natural product **3**. Compound **1** was accordingly identified as 2-propyl dodecanoate, and compound **3** as 3-pentyl tetradecanoate (Fig 3A and 3C).

### Quantitative determination of three esters

ME contained 15.1 ± 4.9 ng of 2-propyl dodecanoate **1**, 323.9 ± 121.2 ng of 3-pentyl dodecanoate **2**, and 20.1 ± 13.1 ng 3-pentyl tetradecanoate **3** on average, whereas FE contained no trace of the corresponding compounds.

### Aggregation activity of the ester

#### Activity of single compounds

Adult male and female beetles were exposed to the identified esters, single compounds **1**–**3**, to screen the function of each compound in the chemical ecology of *L*. *africanus*. The bioassays were performed in closed Petri dishes using a method similar to that of the bioassay involving crude extracts and SiO_2_ column fractions. Synthetic compounds **1**–**3** were prepared in serial doses of 2, 20, 200, 400 and 800 ng/disk. The activity of each single compound against adult *L*. *africanus* beetles is illustrated in Table 3. Adult male and female beetles showed similar responses to all compounds with different doses, excluding compound **2** at the highest dose of 800 ng/disk. Both male and female beetles aggregated in response to the high doses of compound **2**; however the AI values were not high (0.23 ± 0.03 and 0.19 ± 0.03 for female and male beetles, respectively). Generally, female beetles showed a significantly higher preference for treated paper disks than for control paper disks (S3 Table).

**Table 3:** Response of adult *Lyctus africanus* beetles to paper disks treated with synthetic single compounds 1, 2 and 3, as indicated by the aggregation index value (*N* = 20; *n* = 10).

Tested beetles	Doses (ng/disk)	Compound		
		**1**	**2**	**3**
♀	2	0.05 ± 0.04^a(a)^	0.07 ± 0.02^a(b)^	−0.00 ± 0.03^a(a)^
	20	0.08 ± 0.05^a(a)^	0.14 ± 0.02^a(ab)^	0.04 ± 0.05^a(a)^
	200	0.09 ± 0.04^a(a)^	0.17 ± 0.04^a(ab)^	0.09 ± 0.04^a(a)^
	400	0.08 ± 0.03^a(a)^	0.17 ± 0.03^a(ab)^	0.13 ± 0.04^a(a)^
	800	0.04 ± 0.02^b(a)^	0.23 ± 0.03^a(a)^	0.13 ± 0.03^ab(a)^
Tested beetles	Doses	**1**	**2**	**3**
♂	2	−0.02 ± 0.03^a(a)^	0.09 ± 0.05^a(a)^	−0.08 ± 0.04^a(a)^
	20	0.04 ± 0.03^a(a)^	0.05 ± 0.03^a(a)^	0.07 ± 0.02^a(a)^
	200	0.03 ± 0.03^a(a)^	0.05 ± 0.03^a(a)^	−0.04 ± 0.04^a(a)^
	400	−0.01 ± 0.04^a(a)^	0.14 ± 0.03^a(a)^	0.08 ± 0.04^a(a)^
	800	0.01 ± 0.04^b(a)^	0.19 ± 0.03^a(a)^	0.05 ± 0.03^ab(a)^

Notes: Different letters indicate significant differences (*p* < 0.01, Tukey’s HSD test) in the aggregation index (mean ± SEM) between the different doses of the compounds. Letters in parentheses refer to the comparison between female and male beetles for the same dose (Tukey’s HSD test).

#### Activity of three-ester blends

Two blends were used: blends A and B. The mass proportions of blend A were 20:300:20 and those of blend B were 300:300:300. The bioassays suggested that both female and male beetles aggregated more on treated than on control disks (S4 Table). The AI value (Table 4) showed that both female and male aggregation was significantly higher on paper disks treated with blend A than on those treated with blend B (Student’s *t* test, *P* < 0.05).

**Table 4:** Response of adult *Lyctus africanus* beetles to paper disks treated with two different three-ester blends, blend A and B, as indicated by the aggregation index value (*N* = 20; *n* = 10).

Tested beetles	Blend A	Blend B
♀	0.46 ± 0.042*^(ns)^	0.24 ± 0.02*^(ns)^
♂	0.34 ± 0.05*^(ns)^	0.21 ± 0.03*^(ns)^

Notes: Asterisks indicate significant differences (*p* < 0.01, Student’s *t* test) in the aggregation index value (mean ± SEM) within the same sex. Letters in parentheses refer to comparison (by Tukey’s HSD test) between female and male beetles.

ns, not significant.

We evaluated the activity of each natural and synthetic compound (Table 5). As shown by the AI value, the whole natural blend (ME) had the highest ability to induce the aggregation behavior of adult *L*. *africanus* beetles (female: *F* = 29.64, *df* = 39, *P* < 0.0001; male: *F* = 6.08, *df* = 39, *P* = 0.0019). The ester fraction and synthetic blend showed similar effects in eliciting the aggregation behavior of both male and female beetles, whereas any of the synthetic single compound had the lowest tendency to elicit the aggregation behavior.

**Table 5:** Response of adult *Lyctus africanus* beetles to different substances: male crude extract (ME), 10% EtOAc in hexane (ester fraction), synthetic single compound 2, and blend compound, as indicated by the AI value (*N* = 20; *n* = 10).

Tested beetles	ME	Ester fraction	Single compound 2	Blend compound
♀	0.62 ± 0.04^a(^*^)^	0.50 ± 0.03^b(^*^)^	0.23 ± 0.03^c(ns)^	0.46 ± 0.04^b(ns)^
♂	0.36 ± 0.04^a(^*^)^	0.18 ± 0.04^b(^*^)^	0.19 ± 0.03^ab(ns)^	0.34 ± 0.05^ab(ns)^

Notes: Different letters indicate significant differences (*p* < 0.01, Tukey’s HSD test) in the aggregation index value (mean ± SEM) within the same sex. Asterisks in parentheses refer to the comparison between female and male beetles (Student’s *t* test). ns, not significant.

## Discussion

Male and female *L*. *africanus* aggregated as soon as released of beetles in Petri dishes containing paper disks loaded with ME. Furthermore, the beetles frequently stayed on the treated paper disks throughout the 5-min observation period. The rapid recruitment of beetles to ME and their behavior on the treated disks during the bioassay suggest that the compounds are attractants, which acted as aggregation pheromones. In contrast, FE produced insignificant responses in both male and female beetles. A similar result was reported in other genera of Bostrichidae (Coleoptera). Males of the lesser grain borer (*Rhyzopertha dominica*) [20] and larger grain borer (*Prostephanus truncatus*) [21] produce aggregation pheromones and contribute to food sources or breeding site localization [22, 23].

Using GC–MS, ME contained some ester compounds which were recognized as male-specific compounds. Furthermore, hydrocarbons were identified in ME and FE. Further bioassay on separated fractions in an SiO_2_ column confirmed the function of esters in the aggregation behavior of adult *L*. *africanus* beetles. However, the hydrocarbons detected in hexane fraction were not involved in aggregation behavior of *L*. *africanus*.

Chemical analyses revealed that compound **2** was found exclusively in male beetles as a major component. This compound was detected in large amounts (323 ng/beetle), followed by compound **3** (20 ng/beetle) and compound **1** (15 ng/beetle). These amounts may be substantially larger than the actual amounts released by the beetles. A headspace analysis may be useful for detecting actual volatile emissions. However, none of the esters were detected in male *L*. *africanus* using solid-phase micro-extraction sampling (N. Shimizu, personal communication). Another headspace sampling method should be performed to detect the actual volatile emissions released by *L*. *africanus*.

In the present study, synthetic compound **2** was found to induce the aggregation behavior of both female and male beetles at a high dose of 800 ng/disk, higher than its natural amount. However, the responses elicited by the single synthetic compound **2** were not high compared with those by the mixture of natural compounds. It is possible that the activity of single compound **2** was not sufficient to induce the aggregation behavior of *L*. *africanus*. The other single minor compounds **1** and **3** seemed to have no effect on the beetle’s responses when used alone.

Compound **1** is known to be male-specific component of the black larder beetle *Dermestes haemorrhoidali* that acts as an aggregation pheromone [12, 24] and is also a major component of the male abdominal exocrine glands of the black larder beetle *D*. *ater* [25]. This same compound also acts as a minor component of the labial glands of the stingless bee *Trigona corvine* [25]. Compound **3** has not been previously reported.

We found a synergistic effect among the three synthetic esters compounds **1**–**3**, which increased the preference of both male and female beetles for the natural blend. Some insects are aggregated by blends of pheromones consisting of two or more active compounds [24, 26–28]. However, it is still unclear whether synergism was produced by combination of two or three synthetic esters. Further studies are needed to identify the precise combination of esters leading to the highest aggregation response in *L*. *africanus*.

Another finding from our study is the recognition of 3-pentyl dodecanoate (compound **2**) as the major active compound of the aggregation pheromone in *L*. *africanus*. In view of the large amounts produced by male beetles, it is reasonable to assume that this pheromone plays an important role in the ecology of *L*. *africanus*. To the best of our knowledge, this is the first report of 3-pentyl dodecanoate (**2**) as a natural product.

Our study is the first record of pheromone identification in the *Lyctus* beetle. To date, pheromone production in the Bostrichidae family has been found only in two stored-product pests, the lesser and larger grain borers [20, 21]. We believe that our findings will lead to additional research in the development of adult beetle lures and control strategies for Bostrichidae, including *Lyctus*.

## Supporting Information

S1 Fig The experimental design of dual-choice bioassays in a 9-cm Petri dish; (A) and/or (B) two small paper disks for control and treatment. X: the beetle release point.
(TIF)

S2 Fig Structural determination of compound 2 by the comparison of GC retention times among four dodecanoate isomers on a GC capillary column (HP-5 ms): (A) 2-pentyl dodecanoate (2-PD), 2-methylbutyl dodeanoate (2-MBD), 3-methylbutyl dodecanoate (3-MBD), and 3-pentyl dodecanoate (2); (B) Co-chromatography with natural compound 2. (TIF)

S1 Table Aggregation of adult *L*. *africanus* beetles on paper disks treated with the male crude extract (ME), female crude extract (FE), and control, as indicated by the percentage of beetles (*N* = 20; *n* = 10). (DOCX)

S2 Table Aggregation of adult *L*. *africanus* beetles on paper disks treated with some fractions of the male crude extract, as indicated by the percentage of beetles (*N* = 20; *n* = 10). (DOCX)

S3 Table Aggregation of adult *L*. *africanus* beetles on paper disks treated with synthetic compounds 1, 2, and 3, as indicated by the percentage of beetles (*N* = 20; *n* = 10). (DOCX)

S4 Table Aggregation of adult *L*. *africanus* beetles on paper disks treated with two component blends (*N* = 20; *n* = 10). (DOCX)
