# Supplementary figures and images for: Identification of Esters as Novel Aggregation Pheromone Components Produced by the Male Powder-Post Beetle, Lyctus africanus Lesne (Coleoptera: Lyctinae)
Source: PLoS One. 2015 Nov 6;10(11):e0141799. doi: 10.1371/journal.pone.0141799 (PMC4636395; doi:10.1371/journal.pone.0141799)

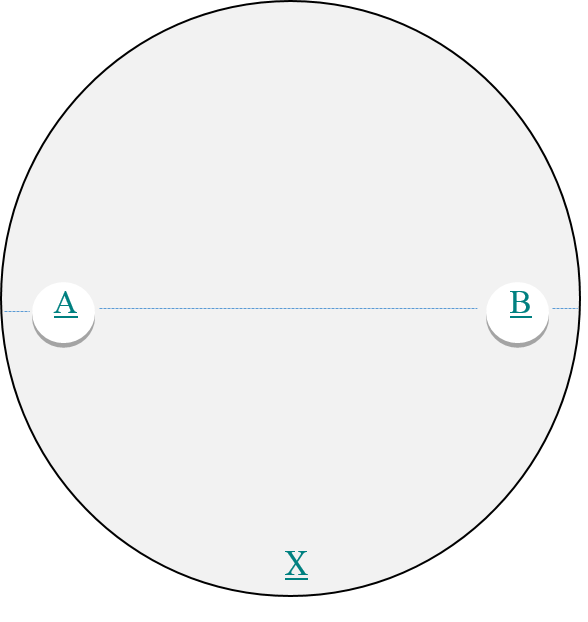

Supplement: S1 Fig — X: the beetle release point. (TIF) [file pone.0141799.s001.tif]

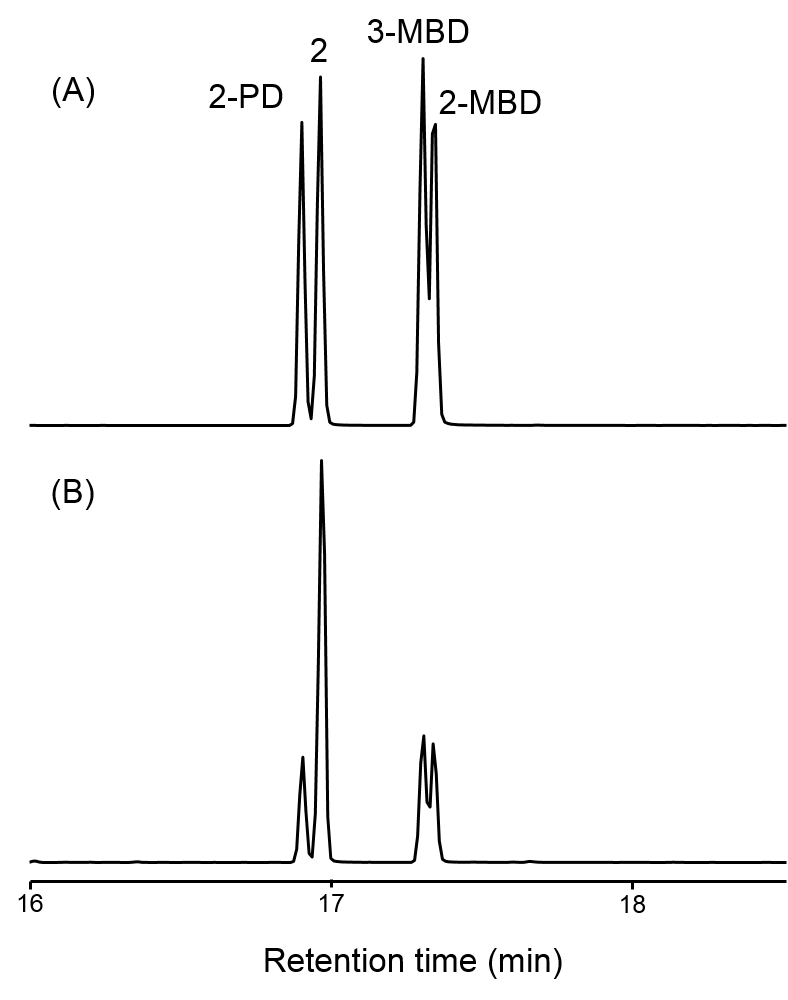

Supplement: S2 Fig — (TIF) [file pone.0141799.s002.tif]
