# Supplementary material for: Identification of Esters as Novel Aggregation Pheromone Components Produced by the Male Powder-Post Beetle, Lyctus africanus Lesne (Coleoptera: Lyctinae)
Source: PLoS One. 2015 Nov 6;10(11):e0141799. doi: 10.1371/journal.pone.0141799 (PMC4636395; doi:10.1371/journal.pone.0141799)
Supplement: S1 Table — (DOCX) [file pone.0141799.s003.docx]

**S1 Table**. Aggregation of adult *L. africanus* beetles on paper disks treated with the male crude extract (ME), female crude extract (FE), and control, as indicated by the percentage of beetles (*N* = 20; *n* = 10).

| Treatment | Tested beetles | % responder beetles | | *P* value |
| --- | --- | --- | --- | --- |
|  |  | Treated | Control |  |
| ME vs. Control | ♀ | 64.00 ± 3.63 | 1.95 ± 0.57 | 0.002* |
|  | ♂ | 42.30 ± 3.40 | 6.40 ± 1.09 | 0.002* |
| FE vs. Control | ♀ | 22.80 ± 4.51 | 16.00 ± 3.08 | 0.432 |
|  | ♂ | 15.05 ± 2.98 | 11.10 ± 1.81 | 0.223 |
|  |  | ME | FE |  |
| ME vs. FE | ♀ | 53.10 ± 3.42 | 2.75 ± 0.72 | 0.002* |
|  | ♂ | 32.70 ± 5.11 | 4.35 ± 1.45 | 0.002* |

Notes: Level of significance difference for number of beetles on disk, shown by asterisk symbols (Matched pairs test).
