# Supplementary material for: Identification of Esters as Novel Aggregation Pheromone Components Produced by the Male Powder-Post Beetle, Lyctus africanus Lesne (Coleoptera: Lyctinae)
Source: PLoS One. 2015 Nov 6;10(11):e0141799. doi: 10.1371/journal.pone.0141799 (PMC4636395; doi:10.1371/journal.pone.0141799)
Supplement: S2 Table — (DOCX) [file pone.0141799.s004.docx]

**S2 Table**. Aggregation of adult *L. africanus* beetles on paper disks treated with some fractions of the male crude extract, as indicated by the percentage of beetles (*N* = 20; *n* = 10).

| Fractions | Tested beetles | % responder beetles | | *P* value |
| --- | --- | --- | --- | --- |
|  |  | Treated | Control |  |
| Hexane | ♀ | 6.83 ± 1.25 | 15.33 ± 1.53 | 0.031* |
|  | ♂ | 2.75 ± 0.80 | 7.83 ± 2.28 | 0.031* |
| 10% EtoAc in hexane | ♀ | 54.85 ± 2.61 | 5.30 ± 1.12 | 0.002* |
|  | ♂ | 31.90 ± 2.82 | 14.45 ± 2.68 | 0.006* |
| 20% EtoAc in hexane | ♀ | 17.60 ± 2.66 | 11.10 ± 2.58 | 0.125 |
|  | ♂ | 17.20 ± 3.27 | 6.30 ± 1.35 | 0.063 |
| 50% EtoAc in hexane | ♀ | 13.10 ± 1.56 | 9.30 ± 1.91 | 0.188 |
|  | ♂ | 3.90 ± 1.26 | 10.40 ± 2.85 | 0.188 |
| EtoAc | ♀ | 8.30 ± 1.62 | 10.20 ± 2.33 | 0.063 |
|  | ♂ | 4.80 ± 0.64 | 5.80 ± 2.26 | 1.000 |

Notes: Level of significant differences between numbers of beetles on disk are shown by asterisks (Matched pairs test).
