# Supplementary material for: Identification of Esters as Novel Aggregation Pheromone Components Produced by the Male Powder-Post Beetle, Lyctus africanus Lesne (Coleoptera: Lyctinae)
Source: PLoS One. 2015 Nov 6;10(11):e0141799. doi: 10.1371/journal.pone.0141799 (PMC4636395; doi:10.1371/journal.pone.0141799)
Supplement: S3 Table — (DOCX) [file pone.0141799.s005.docx]

**S3 Table**. Aggregation of adult *L. africanus* beetles on paper disks treated with synthetic compound **1**, **2**, and **3,** as indicated by the percentage of beetles (*N* = 20; *n* = 10).

| Compound | Tested beetles | Doses (ng/disk) | % responder beetles | | *P* value |
| --- | --- | --- | --- | --- | --- |
|  |  |  | Treated | Control |  |
| **1** | ♀ | 2 | 18.90 ± 3.09 | 13.50 ± 2.27 | 0.232 |
|  |  | 20 | 21.65 ± 4.57 | 13.35 ± 1.75 | 0.186 |
|  |  | 200 | 19.85 ± 2.56 | 11.10 ± 2.16 | 0.018* |
|  |  | 400 | 21.90 ± 3.07 | 14.35 ± 1.66 | 0.027* |
|  |  | 800 | 13.50 ± 1.10 | 9.50 ± 2.02 | 0.074 |
|  | ♂ | 2 | 14.60 ± 2.61 | 16.20 ± 1.48 | 0.537 |
|  |  | 20 | 17.65 ± 2.90 | 13.50 ± 2.72 | 0.432 |
|  |  | 200 | 14.20 ± 1.85 | 11.75 ± 1.97 | 0.264 |
|  |  | 400 | 14.80 ± 2.67 | 15.60 ± 2.39 | 0.540 |
|  |  | 800 | 12.15 ± 3.03 | 11.15 ± 2.03 | 0.748 |
| **2** | ♀ | 2 | 20.15 ± 1.80 | 13.40 ± 1.37 | 0.030* |
|  |  | 20 | 25.40 ± 2.64 | 11.55 ± 1.35 | 0.002* |
|  |  | 200 | 26.25 ± 3.10 | 9.75 ± 1.46 | 0.002* |
|  |  | 400 | 28.75 ± 2.53 | 11.70 ± 1.88 | 0.002* |
|  |  | 800 | 27.95 ± 2.55 | 4.70 ± 0.97 | 0.002* |
|  | ♂ | 2 | 25.05 ± 4.00 | 16.30 ± 1.73 | 0.047* |
|  |  | 20 | 14.70 ± 3.53 | 9.80 ± 1.88 | 0.201 |
|  |  | 200 | 18.30 ± 2.25 | 13.85 ± 2.65 | 0.201 |
|  |  | 400 | 24.15 ± 2.98 | 10.30 ± 1.67 | 0.006* |
|  |  | 800 | 24.3 ± 2.26 | 5.35 ± 1.40 | 0.002* |
| **3** | ♀ | 2 | 16.80 ± 3.15 | 15.95 ± 2.06 | 0.902 |
|  |  | 20 | 16.85 ± 4.31 | 13.40 ± 1.82 | 0.695 |
|  |  | 200 | 21.00 ± 4.12 | 12.05 ± 2.32 | 0.084 |
|  |  | 400 | 23.90 ± 4.18 | 10.95 ± 1.34 | 0.014* |
|  |  | 800 | 18.30 ± 2.58 | 5.50 ± 1.27 | 0.012* |
|  | ♂ | 2 | 10.30 ± 2.25 | 20.70 ± 2.32 | 0.045* |
|  |  | 20 | 16.95 ± 1.96 | 10.15 ± 2.24 | 0.022* |
|  |  | 200 | 12.30 ± 2.97 | 16.50 ± 3.26 | 0.361 |
|  |  | 400 | 22.25 ± 3.41 | 14.80 ± 1.80 | 0.087 |
|  |  | 800 | 13.65 ± 2.25 | 8.65 ± 2.11 | 0.098 |

Notes: Level of significant differences between numbers of beetles on disk are shown by asterisks (Matched pairs test).
