# Supplementary material for: Identification of Esters as Novel Aggregation Pheromone Components Produced by the Male Powder-Post Beetle, Lyctus africanus Lesne (Coleoptera: Lyctinae)
Source: PLoS One. 2015 Nov 6;10(11):e0141799. doi: 10.1371/journal.pone.0141799 (PMC4636395; doi:10.1371/journal.pone.0141799)
Supplement: S4 Table — (DOCX) [file pone.0141799.s006.docx]

**S4 Table**. Aggregation of adult *L. africanus* beetles on paper disks treated with two component blends (*N* = 20; *n* = 10).

| Treatment | Tested beetles | % responder beetles | | *P* value |
| --- | --- | --- | --- | --- |
|  |  | Treated | Control |  |
| Blend A | ♀ | 48.80 ± 4.45 | 2.65 ± 0.82 | 0.002* |
|  | ♂ | 45.50 ± 3.43 | 12.00 ± 2.67 | 0.002* |
| Blend B | ♀ | 27.00 ± 1.97 | 3.45 ± 1.10 | 0.002* |
|  | ♂ | 22.60 ± 3.47 | 1.40 ± 0.43 | 0.002* |

Notes: Level of significant differences between numbers of beetles on disk are shown by asterisks (Matched pairs test).
